# Virgin Coconut Oil Prevents Blood Pressure Elevation and Improves Endothelial Functions in Rats Fed with Repeatedly Heated Palm Oil

**DOI:** 10.1155/2013/629329

**Published:** 2013-06-05

**Authors:** Badlishah Sham Nurul-Iman, Yusof Kamisah, Kamsiah Jaarin, Hj Mohd Saad Qodriyah

**Affiliations:** ^1^Department of Pharmacology, Faculty of Medicine, Universiti Kebangsaan Malaysia, 50300 Kuala Lumpur, Malaysia; ^2^Faculty of Dentistry, Universiti Sains Islam Malaysia, 55100 Kuala Lumpur, Malaysia

## Abstract

This study was performed to explore the effects of virgin coconut oil (VCO) in male rats that were fed with repeatedly heated palm oil on blood pressure, plasma nitric oxide level, and vascular reactivity. Thirty-two male Sprague-Dawley rats were divided into four groups: (i) control (basal diet), (ii) VCO (1.42 mL/kg, oral), (iii) five-times-heated palm oil (15%) (5HPO), and (iv) five-times-heated palm oil (15%) and VCO (1.42 mL/kg, oral) (5HPO + VCO). Blood pressure was significantly increased in the group that was given the 5HPO diet compared to the control group. Blood pressure in the 5HPO + VCO group was significantly lower than the 5HPO group. Plasma nitric oxide (NO) level in the 5HPO group was significantly lower compared to the control group, whereas in the 5HPO + VCO group, the plasma NO level was significantly higher compared to the 5HPO group. Aortic rings from the 5HPO group exhibited attenuated relaxation in response to acetylcholine and sodium nitroprusside as well as increased vasoconstriction to phenylephrine compared to the control group. Aortic rings from the 5HPO + VCO group showed only attenuated vasoconstriction to phenylephrine compared to the 5HPO group. In conclusion, VCO prevents blood pressure elevation and improves endothelial functions in rats fed with repeatedly heated palm oil.

## 1. Introduction

Cardiovascular disease has become the main cause of death worldwide [1]. Hypertension or an increase in blood pressure is among factors that cause cardiovascular complications such as coronary heart disease, atherosclerosis, and stroke [2]. However, an unhealthy lifestyle is the main contributor to the increase in the incidence of hypertension [3]. 

Previous research has shown that heated palm oil causes a significant increase in blood pressure [4]. Hypertension is related to the overproduction of free radicals and lower antioxidant mechanisms in the body [5]. Repeatedly heated palm oil at a high temperature produces free radicals [6]. The presence of antioxidants such as vitamin E is destroyed during the heating process [7]. When the palm oil is heated repeatedly, not only does it generate free radicals but it also reduces antioxidant and vitamin contents, which can lead to oxidative stress. Oxidative stress occurs due to an imbalance between the production of free radicals and a decrease in antioxidant activity in the body. Oxidative stress also leads to low-density lipoprotein (LDL) oxidation [8]. 

Oil that is heated at a high temperature will go through an oxidation process, which causes changes in fatty acid configuration from the cis isomer to the trans. Intake of trans fat correlates with an increase in cardiovascular disease risks [9]. Fatty acids which are oxidized due to repeatedly heated oil cause changes in endothelium function which leads to an impairment in vasodilatation reaction, increase in inflammation and hypertension risks [10] and total serum cholesterol and LDL [6, 11]. Previous research has shown that fried food intake correlates with the decrease in high-density lipoprotein (HDL) level [12].

In this study, repeatedly heated palm oil is used to mimic the situation that happens where people fry foods using the same oil multiple times. This practice is common among Malaysian, as a means to cut expenses. Previous research done by Azman et al. [13] showed that even though night market vendors agreed that repeatedly heated cooking oil is harmful to health, they still continued the practice of using the same cooking oil repeatedly. 

Nowadays, virgin coconut oil (VCO) has become popular due to its beneficial effects. VCO has been shown to have anti-inflammatory, analgesic, and antipyretic properties [14]. VCO has been shown to decrease lipid levels in serum and tissue as well as LDL lipid peroxidation [15]. Consumption of VCO enhances antithrombotic effects related to inhibition of platelet coagulation and low cholesterol level [16]. VCO has been known to have higher antioxidant activity compared to refined coconut oil [17]. It has also been proven that VCO enhances antioxidant activity and inhibits lipid peroxidation in rats [18]. Therefore, it is of great interest for us to investigate whether VCO is able to prevent hypertension in male rats given repeatedly heated palm oil. 

## 2. Materials and Methods 

### 2.1. Animals and Experimental Design

Thirty-two male Sprague-Dawley rats, weighing between 200 and 250 g were obtained from the Laboratory Animal Resource Unit, Universiti Kebangsaan Malaysia. They were randomly divided equally into four groups comprising of eight animals each. The ethical approval for this study was obtained from the Universiti Kebangsaan Malaysia Animal Ethics Committee (PP/FAR/2010/QODRIYAH/14-JULY/309-AUGUST-2010-AUGUST-2011). All animal management and procedures were performed in accordance with the recommended guidelines. 

The rats were kept in stainless-steel cages at room temperature of 27°C ± 2°C with a 12-hour light-dark cycle. All rats had free access to food and water throughout the experiment. After 1 week of acclimatization, each group of rats were fed on the following diets: (i) basal diet (commercial rat chow) (control), (ii) basal diet along with 1.42 mL/kg VCO orally (VCO), (iii) basal diet fortified with 15% weight/weight (w/w) five-times-heated palm oil (5HPO), and (iv) basal diet fortified with 15% weight/weight (w/w) five-times-heated palm oil along with 1.42 mL/kg VCO orally (5HPO + VCO) for 16 weeks. Body weight and food intake were measured weekly. Blood pressure was measured at baseline and at intervals of 4 weeks for a total duration of 16 weeks. Blood samples were collected via access to the orbital sinus prior to treatment and at the end of this study. At the end of the study, the animals were then sacrificed, and thoracic aortas were isolated for measurement of vascular reactivity. 

### 2.2. Virgin Coconut Oil

The VCO used in this study was purchased from Organic Gain Sdn. Bhd., Bandar Baru Bangi, Selangor, Malaysia. It was administered by oral gavage at a dose of 1.42 mL/kg according to the minimal recommended dose of 10 mL per day in humans [19].

### 2.3. Preparation of Diet

The palm oil (Cap Buruh, Lam Soon Edible Oils, Kuala Lumpur, Malaysia) used was purchased from a local store. In this study, the palm oil was heated five times according to the method described by Owu et al. [20]. The heating process involved using 2.5 L of the oil to fry 1 kg of sweet potatoes in a stainless-steel wok. The temperature of the heated oil reached 180°C for 10 minutes. To heat the oil five times, the oil was cooled for 5 hours between heating, and then, the whole frying process was repeated with a new batch of sweet potatoes without any addition of fresh oil. This protocol was in accordance with earlier experimental procedures used in our laboratory [6]. Standard rat chow (Gold Coin, Kepong, Malaysia) was used. The rat chow was ground and mixed with 15% (w/w) of five-times-heated palm oil. The mixture was made into pellets which were dried overnight at room temperature.

### 2.4. Measurement of Blood Pressure

Systolic blood pressure of the prewarmed conscious rats was measured by the tail-cuff method using PowerLab data acquisition systems (ADInstruments, Castle Hill, NSW, Australia). 

### 2.5. Measurement of Plasma Nitric Oxide

Nitric oxide content was indirectly measured by its metabolite nitrite. Blood samples taken were centrifuged to obtain its plasma and kept at −70°C. Plasma samples of 50 *μ*L were put into a 96-well microtiter plate and mixed with 50 *μ*L of modified Griess reagent (Sigma-Aldrich, St. Louis, MO, USA). After 15 minutes of incubation at room temperature in a dark environment, nitrite concentration was measured at 540 nm wave length on an Emax ELISA microplate reader using SoftMax Pro Software (Molecular Devices, Sunnyvale, CA, USA). Nitrite concentration was then determined by plotting a standard curve with increasing concentration of sodium nitrite (Sigma-Aldrich, St. Louis, MO, USA).

### 2.6. Measurement of Vascular Reactivity

The aortic rings were prepared as described by Ajay and Mustafa [21]. The thoracic aorta was dissected, and excess fat and connective tissues were removed. The aorta was cut into ring segments with a width of 3–5 mm and suspended into a 5 mL organ baths containing Krebs solution of the following composition (mM): NaCl 118.0, 2 KCl 4.7, CaCl_2_·2H_2_O 2.5, KH_2_PO_4_ 1.2, MgSO_4_ 1.2, glucose 11.7, NaHCO_3_ 25.0, and EDTA 0.026. The bathing solution was continuously provided with a mixture of oxygen and carbon dioxide. The tissue isometric tension (g) was recorded using a force-displacement transducer (FT03E, Grass Instruments, west Warwick, RI, USA) attached to a MacLab recording system (MacLab model 8 S, ADInstruments, Castle Hill, NSW, Australia). The aortic rings were readjusted to a basal tension of 1 g and allowed to equilibrate over 30 to 45 minutes. During this period, the bathing solutions were replaced every 15 minutes as required.

 Following the equilibration period, the aortic rings were allowed to achieve contractile response to isotonic KCl solution (high K+, 60 mM). Following the washout of responses to high K+, the rings constricted in response to phenylephrine (PE, 10^−7^ M) induced by an addition of acetylcholine (Ach, 10^−5^ M) to assess the endothelial integrity. Only the endothelial intact rings with more than 50% relaxation to Ach were further assessed. In addition, these aortic rings were tested for relaxation responses to increasing concentrations of Ach (10^−10^ M to 10^−5^ M) and sodium nitroprusside (SNP 10^−10^ M to 10^−5^ M) and were recorded in PE (10^−5^ M) precontracted aortic rings. The contractile responses to increasing concentration of PE (10^−10^ M to 10^−5^ M) were also recorded in the rings. Different aortic rings with intact endothelium were used in each experiment. 

### 2.7. Drugs

The drugs used for the vascular reactivity study included acetylcholine chloride, phenylephrine-HCI (Sigma Chemical Co., St. Louis, MO, USA) and sodium nitroprusside (BDH Limited and BDH Laboratory Supplies, Poole, England). 

### 2.8. Statistical Analysis

Results were presented as means ± SEM. Normality of the data was determined using the Shapiro-Wilk test. Statistical differences were determined using the paired Student's *t*-test or one-way ANOVA followed by Tukey's HSD post hoc test to identify the differences using SPSS version 16.0 (SPSS Inc., Chicago, IL, USA). Values of *P* < 0.05 were considered to be significant.

## 3. Results

### 3.1. Body Weight

**Figure 1:**
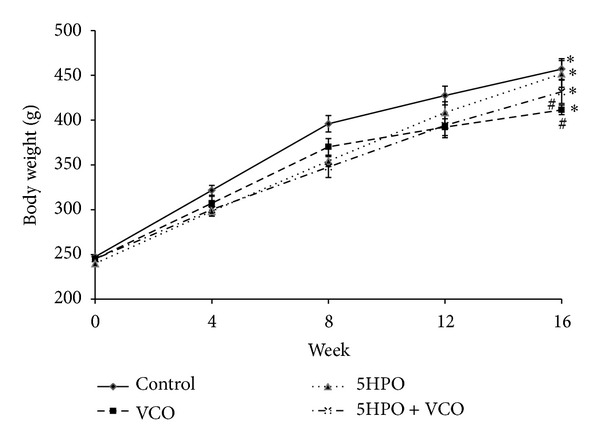
Changes in body weight during the study treatment. Data are expressed as mean ± SEM. VCO, virgin coconut oil; five-times-heated palm oil, 5HPO. *Significant difference (*P* < 0.05) at week 16 compared to week 0 for each group. ^#^Significant difference (*P* < 0.05) compared to control at week 16.

There was a significant increase in body weight of all the groups at week 16 compared to base line. There was no significant difference in body weight of the 5HPO group (451.63 g ± 15.05) compared to the control group (457.00 g ± 11.59). However, body weight in the VCO (411.38 g ± 5.33) and 5HPO + VCO (431.63 g ± 12.97) groups was significantly lower compared to the control group at week 16 (Figure 1). 

### 3.2. Food Intake

**Figure 2:**
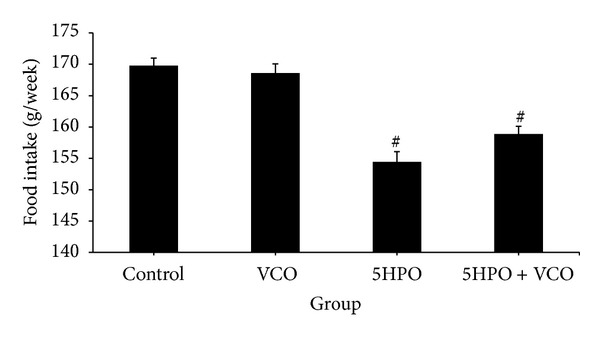
Food intake during the study treatment. Data are expressed as mean ± SEM. VCO, virgin coconut oil; five-times-heated palm oil, 5HPO. ^#^Significant difference (*P* < 0.05) compared to control.

The 5HPO (154.45 g ± 1.62) and 5HPO + VCO (158.86 g ± 1.24) groups showed significantly lower mean food intake compared to the control group (169.77 g ± 1.21). There was no significant difference in food intake in the VCO (168.59 g ± 1.46) compared to the control group. There was also no significant difference of food intake in the 5HPO + VCO compared to the 5HPO group (Figure 2). 

### 3.3. Blood Pressure

**Figure 3:**
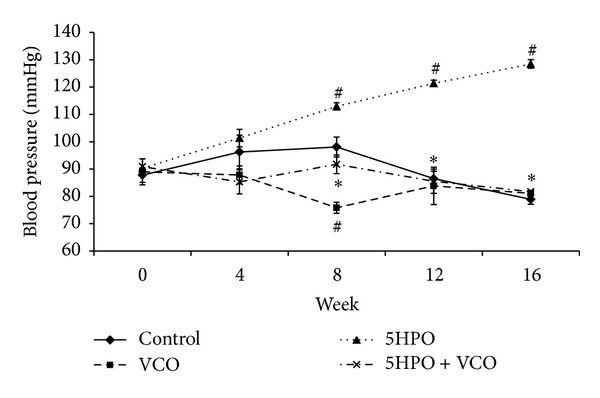
Changes in blood pressure during the study treatment. Data are expressed as mean ± SEM. VCO, virgin coconut oil; five-times-heated palm oil, 5HPO. ^#^Significant difference (*P* < 0.05) compared to control. *Significant difference (*P* < 0.05) 5HPO + VCO compared to group 5HPO.

Starting from week 8 to week 16, the 5HPO group showed a significant increase in blood pressure compared to the control group. Blood pressure in the 5HPO + VCO group is significantly lower compared to the 5HPO group from week 8 to week 16. Blood pressure in the VCO group (75.83 mmHg ± 1.99) is significantly lower compared to the control group (98.08 mmHg ± 3.61) at week 8 only (Figure 3). 

### 3.4. Changes in the Plasma Nitric Oxide Metabolite Level

**Figure 4:**
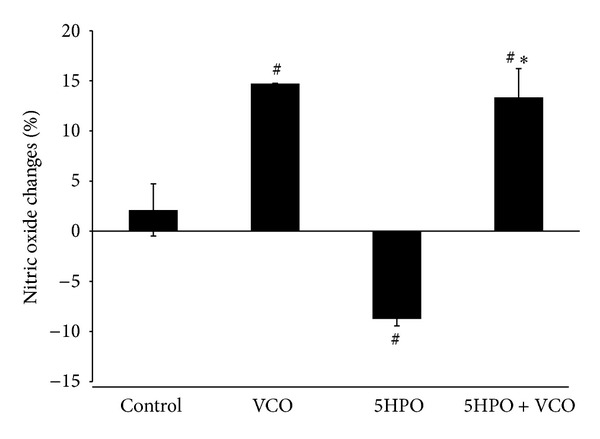
Changes in plasma nitric oxide metabolites during the study treatment. Data are expressed as mean ± SEM. VCO, virgin coconut oil; five-times-heated palm oil, 5HPO. ^#^Significant difference (*P* < 0.05) compared to control. *Significant difference (*P* < 0.05) 5HPO + VCO compared to group 5HPO.

The 5HPO group (−8.75% ± 0.7) showed a significant decrease in nitric oxide level compared to the control group (2.12% ± 2.6). The VCO (14.73% ± 0.02) and 5HPO + VCO (13.36% ± 2.86) groups showed an increase in nitric oxide level compared to the control group. For the 5HPO + VCO group, there was a significant increase in nitric oxide level compared to the 5HPO group (Figure 4). 

### 3.5. Vascular Response

#### 3.5.1. Relaxation in Response to Acetylcholine (Ach)

**Figure 5:**
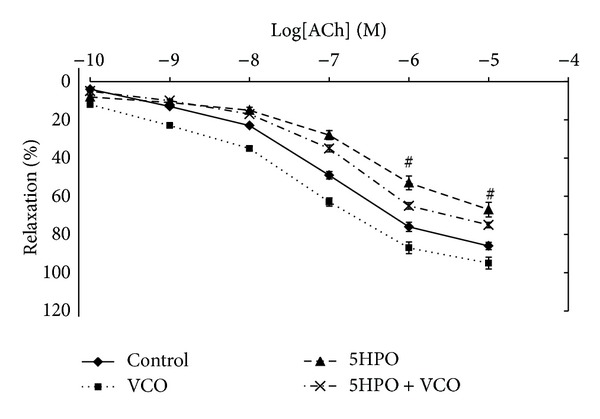
Endothelium-dependent relaxation in response to acetylcholine in aortic rings isolated from rats fed with basal diet (control), virgin coconut oil (VCO), five-times-heated palm oil (5HPO), and five-times-heated palm oil along with VCO (5HPO + VCO) at different concentrations. Data are expressed as mean ± SEM. ^#^Significant difference (*P* < 0.05) compared to control.

The percentage of relaxation at 10^−5^ M and 10^−6^ M concentration for 5HPO group was significantly lower compared to the control group. There was no significant difference in the percentage of relaxation in the 5HPO + VCO group compared to the 5HPO group at all concentrations (Figure 5). 

#### 3.5.2. Relaxation in Response to Sodium Nitroprusside (SNP)

**Figure 6:**
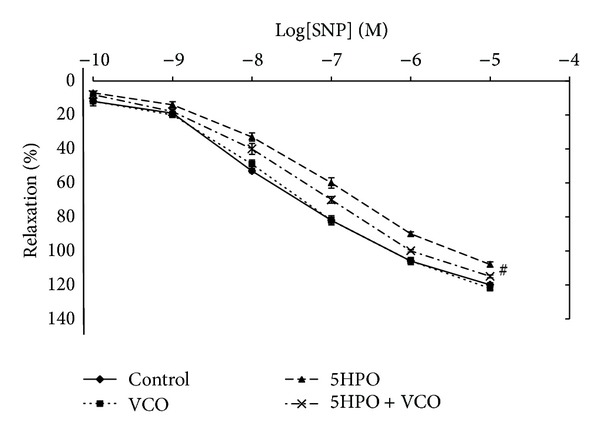
Endothelium-independent relaxation in response to sodium nitroprusside in aortic rings isolated from rats fed with basal diet (control), virgin coconut oil (VCO), five-times-heated palm oil (5HPO), and five-times-heated palm oil along with VCO (5HPO + VCO) at different concentrations. Data are expressed as mean ± SEM. ^#^Significant difference (*P* < 0.05) compared to control.

Vasodilatation in response to the highest tested concentration (10^−5^ M) was significantly attenuated in the aortic ring obtained from the 5HPO (108% ± 1.55) group compared to the control group (120% ± 1.25). There was no significant difference in vascular relaxation response in the 5HPO + VCO group (115% ± 0.84) compared to the 5HPO group (Figure 6).

#### 3.5.3. Contractile Response to Phenylephrine (PE)

**Figure 7:**
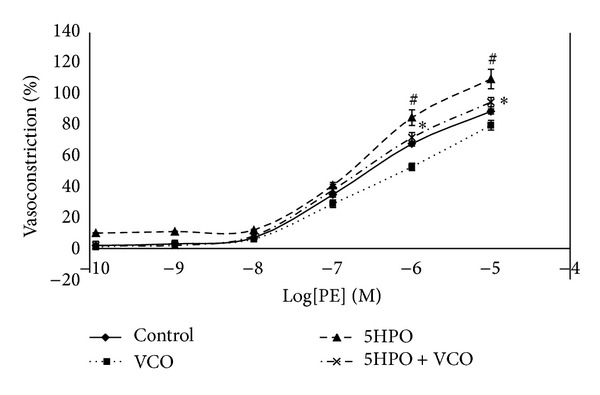
Contractile response induced by phenylepinephrine in aortic rings isolated from rats fed with basal diet (control), virgin coconut oil (VCO), five-times-heated palm oil (5HPO), and five-times-heated palm oil along with VCO (5HPO + VCO) at different concentrations. Data are expressed as mean ± SEM. ^#^Significant difference (*P* < 0.05) compared to control. *Significant difference (*P* < 0.05) 5HPO + VCO compared to group 5HPO.

The vasoconstriction in response towards PE was significantly augmented in the aortic rings from the 5HPO group compared to the control group at a concentration of 10^−6^ to 10^−5^ M. The aortic rings from the 5HPO + VCO group showed a significant decrease in vasoconstriction compared to the 5HPO group at a concentration of 10^−6^ to 10^−5^ M (Figure 7). 

## 4. Discussion

In this study, it was found that food intake in the repeatedly heated oil diet was lower compared to the control group. However, we observed that there was no significant difference in body weight between the 5HPO diet and the control group at week 16. Similar results were also obtained from a study done by Leong et al. [22], using repeated heated oil. Even though the food intake for the VCO and control diet was similar, the VCO diet group was found to have a decreased body weight compared to the control group at week 16. Food intake in the 5HPO + VCO diet was the same as 5HPO diet, but rats from the 5HPO + VCO diet group experienced a reduction in body weight compared to the control group at week 16. This shows that VCO supplementation causes a decrease in body weight. Previous studies in humans have shown that VCO appears to promote a reduction in abdominal obesity [23, 24]. According to a study conducted by St-Onge [25], medium-chain fatty acids (MCFA), compared to long-chain fatty acids, increase energy expenditure and resulted in faster satiety. MCFA found in VCO may aid as a beneficial replacement for other fats in the diet to help promote fullness and also increase caloric expenditure. The fat content helps to slow down the emptying of the stomach. Apart from that, MCFA are also directly broken down and transported to the liver as fuel. Therefore, VCO is utilized for energy and is less likely to get stored as fat, and this probably explains why it is able to reduce abdominal obesity in humans and body weight in study animals. 

Oil that is heated repeatedly contains more saturated fatty acids than unsaturated fatty acids [22]. The body weight increment in rats in the 5HPO diet is not significantly different compared to the control group even though their food intake is lower because saturated fatty acid intake is capable of increasing tissue adiposity. The mechanism involved is due to the decrease of hormone-sensitive lipase and sympathetic activity in adipose tissue. Peroxisome proliferator-activated receptor (PPAR), which is a transcription expression factor, plays a role in proliferation and adiposity differentiation that causes adiposity apoptosis. It is then influenced by saturated fatty acids which lead to adiposity increment (fat tissue growth) that causes body weight increment [26]. 

The 5HPO diet causes blood pressure elevation. This study shows that feeding with repeatedly heated palm oil causes harm to health in rats by increasing hypertension risk. Previous research also showed that an increment in blood pressure occurs in rats that are administered oxidized oil [6, 27]. This is probably due to the increase in oxidative stress, thus causing changes in the nitric oxide level [6]. Reactive oxygen species (ROS) play an important role in the formation of hypertension [28], and ROS are formed during the heating process. Overproduction of ROS is capable of impairing cells and causing blood pressure elevation. The cell membrane is a structure that is sensitive towards oxidative attack due to the high content of polyunsaturated fatty acid (PUFA). Blood pressure elevation is due to the production of free radicals that decreases the NO level [29]. 

VCO supplementation prevents the blood-pressure-raising effect of 5HPO. The blood-pressure-lowering effect of VCO may be attributable to its high polyphenol component [17, 18]. Previous studies have also shown that polyphenol was able to reduce blood pressure in hypertensive subjects [30, 31]. Rats on the VCO diet alone had a transient reduction in blood pressure only at week 8 compared to control. A study by Diebolt et al. [32] showed that short-term administration of polyphenolic compounds reduced blood pressure in normotensive rats. They hypothesized that polyphenol was able to stimulate NO released from the endothelium, giving rise to vasodilation and blood pressure reduction. The reason for the short-term effect is unclear because plasma nitric oxide level was taken at the start and end of the study, not monthly as with the blood pressure measurements. The polyphenol component has also been shown to prevent LDL oxidation, and a diet that contains VCO supplementation increases antioxidant status in rats [15, 18]. The cholesterol level and other lipid parameters in tissue and serum maintained within the normal range as well as increment in HDL concentration due to polyphenol contents in VCO [15]. 

Intake of the 5HPO diet is found to reduce plasma NO level significantly. Overproduction of free radicals increases inactivation of NO which leads to reduction of NO bioavailability. Hence, a reduction of NO causes blood pressure elevation. Intake of VCO diet shows plasma NO level higher than the control group. It also increases plasma NO levels in rats fed with the 5HPO diet. This is thought to be due to the antioxidant component polyphenol, found in VCO which is responsible for increasing NO bioavailability. Antioxidant contents in VCO are possibly capable of providing protection effects by reducing oxidative stress and thus maintaining the NO bioavailability [33]. 

In this study, it was found that the relaxation induced by acetylcholine-dependent endothelium was attenuated in rats fed with the 5HPO diet. Release of NO plays a role in determining the balance between vascular smooth muscle relaxation and vasoconstriction. If NO bioavailability decreases, this then leads to attenuation of vascular smooth muscle relaxation and vasoconstriction [34]. The antioxidant protective effects in oil probably deteriorate when palm oil is repeatedly heated. 

The SNP is an endothelial vascular vasodilatation agent. SNP induces relaxation by releasing NO into the tissue, while the breakdown of the SNP molecule produces NO which activates guanylate cyclase to increase the formation of cyclic diguanylate monophosphate, which causes vascular smooth muscle relaxation. However, relaxation induced by SNP-independent endothelium was attenuated in rats that were given the 5HPO diet compared to the control group at the highest concentration of 10^−5^ M. This is maybe due to the reduced bioavailability of NO which is involved in vasodilatation [34]. 

The rats on the 5HPO + VCO diet showed attenuated vasoconstriction induced by phenylephrine-dependent endothelium compared to the 5HPO diet. Free radicals such as anion superoxide are related to the increase in vascular reactivity including vasoconstriction [35]. The blood-pressure-raising effect of this study was similar to previous research which showed that repeatedly heated oil causes attenuation in vascular reactivity [4]. Repeatedly heated oil also produces toxic products which can increase blood pressure, thus interrupting the endothelium balance. Vitamins and foods that contain antioxidants are capable of improving vascular reactivity and decreasing the bad effects on blood vessels which could prevent hypertension, thus maintaining the endothelium balance. VCO supplementation is capable of improving endothelial function because it is rich in antioxidants. 

## 5. Conclusion

This study showed that VCO supplementation is capable of preventing elevation in blood pressure and also decreasing deactivation of nitric oxide in male rats fed with repeatedly heated palm oil. In addition, VCO does not influence relaxation but decreases vasoconstriction of the endothelium.
